# Age-related differences in visual encoding and response strategies contribute to spatial memory deficits

**DOI:** 10.3758/s13421-020-01089-3

**Published:** 2020-08-31

**Authors:** Vladislava Segen, Marios N. Avraamides, Timothy J. Slattery, Jan M. Wiener

**Affiliations:** 1 Ageing and Dementia Research Centre, Bournemouth University, Bournemouth, UK; 2 Department of Psychology, Bournemouth University, Bournemouth, UK; 3 Department of Psychology, University of Cyprus, Nicosia, Cyprus; 4 Rise Nicosia, Cyprus

**Keywords:** Aging, Decision making, Eye movements, Spatial cognition, Perception

## Abstract

Successful navigation requires memorising and recognising the locations of objects across different perspectives. Although these abilities rely on hippocampal functioning, which is susceptible to degeneration in older adults, little is known about the effects of ageing on encoding and response strategies that are used to recognise spatial configurations. To investigate this, we asked young and older participants to encode the locations of objects in a virtual room shown as a picture on a computer screen. Participants were then shown a second picture of the same room taken from the same (0°) or a different perspective (45° or 135°) and had to judge whether the objects occupied the same or different locations. Overall, older adults had greater difficulty with the task than younger adults although the introduction of a perspective shift between encoding and testing impaired performance in both age groups. Diffusion modelling revealed that older adults adopted a more conservative response strategy, while the analysis of gaze patterns showed an age-related shift in visual-encoding strategies with older adults attending to more information when memorising the positions of objects in space. Overall, results suggest that ageing is associated with declines in spatial processing abilities, with older individuals shifting towards a more conservative decision style and relying more on encoding target object positions using room-based cues compared to younger adults, who focus more on encoding the spatial relationships among object clusters.

**Electronic supplementary material:**

The online version of this article (10.3758/s13421-020-01089-3) contains supplementary material, which is available to authorized users.

## Introduction

The ability to recognise a place from different perspectives is crucial for everyday functioning. It requires remembering the locations of objects relative to each other or relative to the environment (Epstein, Harris, Stanley, & Kanwisher, 1999), and depends on the binding of the memory for object identity with the memory for its location (Postma, Kessels, & van Asselen, 2008; Waller, 2006). The quality of such spatial representations depends on the resolution with which spatial information is encoded (Cowell, Barense, & Sadil, 2019; Ekstrom & Yonelinas, 2020). A coarse spatial representation, for example, may only contain the categorical positions of the objects, such as “the door is in the far right of the room”. Fine-grained representations, in contrast, contain precise metric information about the locations of objects (Evensmoen, Lehn, Witter, Nadel, & Håberg, 2013).

Once a spatial representation of a place is created, visual, vestibular and proprioceptive inputs during active movement can be used to update the representation to allow place recognition from a different perspective (Christou & Bülthoff, 2000; Waller, Montello, Richardson, & Hegarty, 2002). However, if physical movement is absent, recognition across different perspectives can be achieved through the formation of a viewpoint-independent representation or by mental manipulations of the new or stored representation (Holmes, Newcombe, & Shipley, 2018; King, Burgess, Hartley, Vargha-Khadem, & O’Keefe, 2002; Klencklen, Després, & Dufour, 2012). Possible manipulations include: (1) mentally rotating the new representation in alignment with the stored representation, (2) imagining moving around, and (3) rotating the stored representation to match the representation viewed from the current perspective (Hegarty & Waller, 2004; King et al., 2002). Hereafter, we refer to these mental transformations collectively as spatial-perspective taking (Hegarty & Waller, 2004).

Neuroimaging research suggests that the hippocampal circuit and the retrosplenial cortex support the computations involved in spatial-perspective taking (King et al., 2002; Vargha-Khadem et al., 1997). The hippocampus may also allow place recognition across different perspectives by enabling the development of viewpoint-independent representations of the environment (Goodrich-Hunsaker & Hopkins, 2010; Hartley, Maguire, Spiers, & Burgess, 2003; Morris, Garrud, Rawlins & O’Keefe, 1982; Wolbers & Büchel, 2005). Furthermore, the hippocampus is involved in object-location binding (Zimmermann & Eschen, 2017) and the binding of high-resolution perceptual information, including spatial information (Kolarik et al., 2016), into a single representation (Erez, Lee, & Barense, 2013). Together, these studies demonstrate that the hippocampus plays an important role in development of flexible fine-grained spatial representations and the processes involved in place recognition across different perspectives.

Several studies have shown that the hippocampal circuit is particularly vulnerable to age-related alterations (Antonova et al., 2009; Lester, Moffat, Wiener, Barnes, & Wolbers, 2017; Meulenbroek, Petersson, Voermans, Weber, & Fernández, 2004; Moffat, Kennedy, Rodrigue, & Raz, 2007). Thus, it is not surprising that ageing is associated with declines in spatial memory (Hartley et al., 2007; Montefinese, Sulpizio, Galati, & Committeri, 2015; Muffato, Hilton, Meneghetti, De Beni, & Wiener, 2019). Muffato et al. (2019) investigated the nature of spatial memory deficits in ageing by presenting participants with images of places defined by the spatial arrangement of four different objects in an open field. At test, the places were presented from different perspectives and participants decided whether the place was the same or different to that seen during encoding. Age-related performance deficits were found when objects within a scene swapped positions but not when they were substituted with new objects. This highlights a specific age-related deficit in binding the remembered objects to their locations, whilst object-identity memory remained relatively intact in ageing (cf. Allison & Head, 2017; Cushman, Stein, & Duffy, 2008; Head & Isom, 2010).

As Muffato et al. (2019) did not parametrically manipulate the amount of spatial change within the scene, it remains unclear if cognitive ageing also affects the resolution with which spatial representations are formed. That is, older adults may experience difficulties in forming detailed, fine-grained spatial representations, therefore relying more on coarser representations compared to younger adults. This idea is consistent with findings from a spatial working-memory study in which older participants were able to memorise the coarse position of objects on a computer screen, but were less precise than younger participants (Nilakantan, Bridge, VanHaerents, & Voss, 2018). The authors proposed that age-related hippocampal neurodegeneration could explain the difficulties in forming fine-grained representations. This interpretation is in line with patient research showing that young patients with hippocampal damage can form coarse memories of environments but have problems identifying the precise locations of previously encoded objects (Kolarik et al., 2016; Kolarik, Baer, Shahlaie, Yonelinas, & Ekstrom, 2018). Given that ageing is associated with hippocampal atrophy (Moffat et al., 2007), we expect spatial memory to be less fine-grained in older individuals than in young adults. To our knowledge this has not yet been demonstrated empirically.

There is currently no consensus on how ageing affects spatial-perspective taking. Some studies showed that perspective shifts resulted in similar performance declines in young and older adults (e.g., Muffato et al., 2019), while other studies have reported specific age-related deficits in perspective-taking abilities (Inagaki et al., 2002; Montefinese et al., 2015; Watanabe, 2011). It thus remains unclear whether there is a specific age-related deficit in spatial-perspective taking over and above general age-related slowing and cognitive decline.

Here we present an exploratory study combining eye-tracking and diffusion modelling to study age-related differences in the ability to recognize spatial configuration across different perspectives. Similar to earlier studies (Montefinese et al., 2015; Muffato et al., 2019), participants encoded object positions from one perspective and then reported if the objects were in the same or different positions when presented with the scene from a new perspective. To investigate age-related differences in the resolution of spatial representations, we manipulated the spatial arrangement of objects in two different ways: we either changed the precise position of objects within the spatial arrangement between encoding and test so that participants would need to employ fine-grained spatial knowledge to respond correctly, or we introduced a change in the whole spatial arrangement that could be detected using a coarser representation. We unpacked the processes involved in decision making using diffusion modelling, which assumes that decisions are based on evidence that is accumulated over time (Ratcliff & Rouder, 1998). Diffusion modelling combines response times and accuracy to estimate a number of parameters, including response bias (tendency to classify stimuli more as ‘same’ or ‘different’), response boundaries (the amount of information needed to make a decision), drift rate (the rate of information accumulation), and the time required to execute the motor response (Ratcliff, Smith, Brown, & McKoon, 2016).

In ageing research, traditional response-time analyses are complicated by age-related delays in non-decisional processes such as visual processing speed and response execution (Owsley, 2011; Ren, Wu, Chan, & Yan, 2013). This may lead to the incorrect conclusion that ageing is associated with processing deficits and may discourage researchers from using response times in their analysis (e.g. Hartley et al., 2007; Muffato et al., 2019), despite the informative value of this measure in identifying decisional styles in particular speed-accuracy trade-offs. Diffusion modelling can overcome this by modelling separately task-specific information processing (i.e. performance), decisional styles that depend on response conservativeness, and non-decisional processes. By doing so it provides a cleaner measure of the information-processing efficiency (drift rate) whilst allowing the investigation of speed-accuracy trade-offs using a single parameter – response boundaries (Ratcliff et al., 2016; Voss, Nagler, & Lerche, 2013). This is particularly relevant to ageing research in which the patterns of accuracy and response times often differ across age groups (Ratcliff, Thapar, & McKoon, 2006a, 2006b; Watanabe & Takamatsu, 2014).

In tasks with a memory component, drift rate typically represents the quality of the match between the memory trace and the test stimuli (Ratcliff et al., 2004a, b; Spaniol, Madden, & Voss, 2006; White, Ratcliff, Vasey & McKoon, 2009). For example, in word-recognition tasks, words that are more strongly encoded result in higher drift rates (Ratcliff et al., 2004a, b), whilst deficits in episodic memory lead to reduced drift rates (Spaniol et al., 2006). In other words, drift rates depend on the ability to accurately encode information and to access the corresponding representation at test. Drift rates are independent from non-decisional processing and decision styles. In the current task, participants needed to encode the locations of objects in the environment, and access and compare those representations at test following a perspective shift to determine if the objects were in the same or different positions. Thus, drift rate represents participants’ ability to encode the locations of objects in the environment and to access and manipulate these representations after a perspective shift (Hegarty & Waller, 2004).

In addition to collecting accuracy and response-time measures, we used eye-tracking to further investigate potential age-related changes in the encoding of spatial relationships. Past research demonstrates that gaze behaviour is sensitive to the strategies adopted in solving spatial tasks (Schmidt et al., 2007). For example, Livingstone-Lee et al. (2011) showed that the environmental features participants gazed at in the first second of a navigation trial allowed distinguishing between different navigation strategies. Similarly, Bécu, Sheynikhovich, Tatur, Agathos, Bologna, Sahel and Arleo (2019) showed that gaze dynamics are predictive of the spatial cue preferences that participants use to anchor their spatial representations. Here, we rely on eye-tracking data to also delineate the automatic processes that may influence encoding strategies (Schütt, Rothkegel, Trukenbrod, Engbert, & Wichmann, 2019).

Although encoding strategies have not yet been investigated in place recognition, some navigation studies suggest that ageing is associated with changes in encoding of spatial information. For example, Grzeschik, Conroy-Dalton, Innes, Shanker, and Wiener (2019) report that older adults spent less time than younger adults looking at unique, navigationally relevant, landmarks during route learning. Also, Bécu et al. (2019) reported that older adults engage less in explorative gaze behaviour when reorienting during real-world navigation when compared to young adults. These age-related changes in visual-encoding strategies may also be relevant to our task. Specifically, participants need to ‘reorient’ after a perspective shift in order to solve the task. This reorientation likely involves attending to the same ‘relevant’ environmental cues during encoding and test.

Given that age-related differences during spatial encoding in tasks similar to the one presented here have not been previously investigated, we adopted an exploratory approach to the analysis of gaze behaviour. If differences in encoding strategies contributed to age-related differences in spatial memory, we expect systematic differences across several gaze parameters between younger and older adults and correlations between gaze parameters and behavioural performance.

With respect to the behavioural results, we expected to replicate earlier findings showing greater difficulties with spatial memory in older adults, to observe declining performance with increasing perspective shift, and to find lower performance in trials that require fine-grained spatial knowledge than in trials that can be solved using coarser representations. Finally, if older adults have greater difficulties than younger adults in encoding fine-grained spatial information, we expected an interaction between age group and condition, with older adults showing greater performance reduction in trials that require fine-grained spatial knowledge.

For the diffusion-modelling analysis, the key prediction is that drift rates would be lower in older compared to younger participants. In addition, we predicted that older adults would be more conservative in their responses, which would be reflected in wider response boundaries. This prediction is based on research from other cognitive domains showing age-related widening of response boundaries (recognition memory: Spaniol et al., 2006; perceptual learning: Ratcliff et al., 2006a, 2006b; language: Ratcliff et al., 2004a, b). Furthermore, ageing is associated with a greater tendency to identify novel places as familiar as a result of a pattern-completion bias (Vieweg, Stangl, Howard & Wolbers, 2015). We, therefore, expected older adults to show a greater bias towards responding that stimuli are the same even if a change was introduced. Lastly, since ageing is associated with reductions in motor speed (Ren et al., 2013) and visual function (Owsley, 2011) we expect longer non-decision response rates in older than younger participants.

## Method

### Participants

Thirty-eight young (mean age = 21.82 years, SD = 6.92; age range = 18–31 years; 23 females and 15 males) and 38 adults aged 60 years and over (mean age = 70.1 years, SD = 4.79, age range = 60–83 years; 23 females and 15 males) took part in this study. Participants were recruited either through Bournemouth University’s participant recruitment system or through opportunity sampling in the community. Older adults received monetary compensation for their time. Younger participants received either course credits or monetary compensation. Participants were screened for mild cognitive impairment using the Montreal Cognitive Assessment (MoCA; Nasreddine et al., 2005). Based on a threshold score of 23/30 (Luis, Keegan, & Mullan, 2009; Waldron-Perrine & Axelrod, 2012), no participants were excluded from the final analyses. All participants gave their written informed consent in accordance with the Declaration of Helsinki (World Medical Association, 2013).

Given the reports of sex differences in navigation and spatial cognition (Coutrot et al., 2019; Mueller, Jackson, & Skelton, 2008), we first ran an exploratory analysis focusing on sex, but did not find any performance differences between sexes (see Online Supplementary Materials). As the current study was not designed to investigate sex differences, we did not include sex as a factor in any further analyses.

## Materials

### Virtual environment

**Fig. 1:**
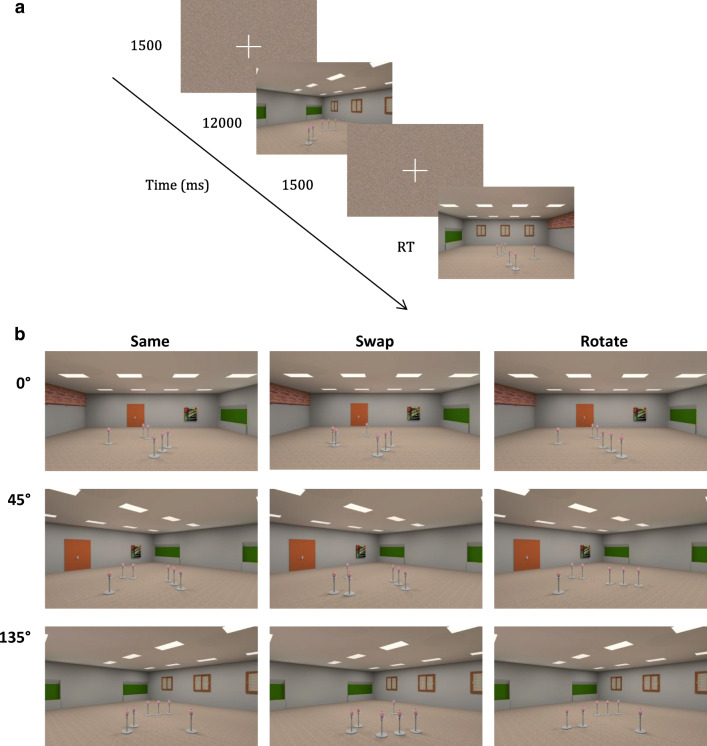
(**A**) Experimental protocol. (**B**) Examples of experimental stimuli for different conditions (*Same*, *Swap* and *Rotate*) and perspective shift (*0°, 45°, 135°)*

The virtual environment was designed using SketchUp Make 2017 (Trimble Inc., 2017) and depicted a rectangular room (13.5 m x 14.6 m) that contained visual cues on the walls including a door, windows, and a painting. The room also contained six identical objects – pink vases on metal stands – that were placed in the centre of the room (Fig. 1).

The experimental stimuli were renderings of the environment from eight different viewpoints with a horizontal field of view of 50°. These viewpoints were arranged at 45° intervals on a circle with a radius of 6.5 m surrounding the target objects (Fig. 1). The objects were arranged in clusters of one, two and three objects. The cluster positions within the room were changed to provide six unique configurations that were used in the experiment. Stimuli were presented with OpenSesame 3.1.7 (Mathôt, Schreij, & Theeuwes, 2012) and a standard computer keyboard was used to record responses.

### Eye-tracking recording

Eye movements were recorded using an Eyelink II (SR Research) head-mounted eye-tracker at a rate of 500 Hz. Calibrations were performed at least three times and drift correction was performed prior to each trial. The experiment was presented on a 102-cm screen (diagonal) with an aspect ratio of 16:9 and a resolution of 1,920 x 1,080 pixels. Participants were seated 100 cm from the monitor. The physical horizontal field of view of the screen at this distance was 47.7°.

### Design

The experiment followed a mixed 2 (Age Group: young vs. older adults) × 3 (Condition: *Rotate, Same, Swap*) x 3 (Perspective Shift: *0°, 45°, 135°*) design with Condition and Perspective Shift manipulated within participants.

### Procedure

Both younger and older adults completed the MoCA before taking part in the experiment. To familiarise participants with the virtual environments, we asked them to watch a 24-s video clip providing a 360° overview of the virtual room without the objects.

Each experimental trial started with a fixation cross and a scrambled stimuli mask (1,500 ms). In the subsequent learning phase, participants were presented with a rendering of one of the six unique configurations of the objects from one of the eight possible viewpoints (48 different renderings) for 12 s. After this learning phase, participants were again presented with a fixation cross and a scrambled stimuli mask for 1,500 ms (Fig. 1A). In the test phase, participants were presented with a rendering of the room either from the same viewpoint (*0°*) or from a different viewpoint that involved a *45°* or *135°* perspective shift. Each perspective level (*0°, 45°,* and *135°*) was used in a third of all trials.

Participants’ task was to decide whether or not the locations of the objects (the pink vases) in the test phase were identical to those in the learning phase. In 50% of the trials the objects remained in the same locations, whilst they moved between learning and test in the remaining 50% of the trials. Specifically, the locations of the objects were changed either by swapping the locations of two of the three clusters (*Swap* condition) or by rotating the cluster consisting of two or three objects by 60° (*Rotate* condition, Fig. 1B). While the *Swap* manipulation changed the whole spatial arrangement and could be detected using coarse spatial representation, the *Rotate* manipulation was more subtle as it maintained the overall configuration of objects and required a fine-grained spatial representation. It should be noted that the cluster consisting of one object was never rotated, as this would not yield a change in the position/orientation of that cluster.

The experiment consisted of 192 experimental trials presented in randomised order and preceded by ten practice trials. The entire study took around 2 hours to complete and participants were free to take breaks when they wished. Overall, 96% of our participants completed the entire study with two older adults withdrawing from the experiment after completing 144 trials and one younger adult after completing 168 trials.

### Data analysis

Stastistical analyses were carried out using R (R Core Team, 2013) with the exception of diffusion modelling, which was carried out using fastDM (Voss & Voss, 2007). The parameters that were obtained from diffusion modelling (drift rate, response conservativeness, non-decision response times) as well as behavioural data (d’ and Bias, sdt.rmcs package in R; Todorova, 2017) were analysed with linear mixed-effects models (LME) using LME4 (Bates, Kliegl, Vasishth, & Baayen, 2015) in R (R Core Team, 2013). For the d’ and the bias LMEs we defined the contrasts as follows: Age Group and Condition (*Rotate/Swap*) were coded using effect coding; Perspective Shift was defined as successive difference contrasts (MASS package in R; Venables & Ripley, 2002) so that the *0°* was compared to *45°* and *45°* was compared to *135°*. For drift rate and boundary separation analysis the same contrasts were used for Age Group and Perspective Shift whilst Condition (*No Change/Rotate/Swap*) was coded using treatment coding with the *No Change* condition as the baseline. Age Group, Perspective Shift and Condition were used as fixed factors across all LMEs. All models included the maximal random effects structure justified by the design: for d’ a random by-subject intercept and slope for Condition and Perspective Shift (no interaction) were used. For drift rate and boundary separation analysis only a random by-subject intercept was used.

Differences between age groups in gaze parameters, non-decision response times and starting bias were examined using the Bootstrap-t method (5,000 resampling) with 20% trimmed means (Wilcox & Keselman, 2003). This method provides a more robust estimation of central tendency than a standard t-test as it reduces the probability of type 1 error and bias and does not compromise power as compared to median-based methods (Wilcox & Keselman, 2003).

To estimate the parameters of the diffusion model we used the Kolmogorov-Smirnov (KS) test statistic *T* (Kolmogoroff, 1941) as the optimization criterion in an iterative search for the best-fitting model solution (Voss, Voss, & Lerche, 2015). We estimated the drift rate (*v*) and response conservativeness for each participant across each experimental condition (Perspective Shift [0°, 45°, 135°] and Condition [*Swap, Rotate*]). We also estimated the starting point bias (*z*) for each participant and the non-decision response time (*t0*). Based on the procedure suggested by Voss et al. (2013), outliers were removed from the individual response-time distributions using the interquartile range method. This allowed estimating the specified parameters for 37 young adults and 36 older adults.

## Results

### Behavioural data

**Fig. 2:**
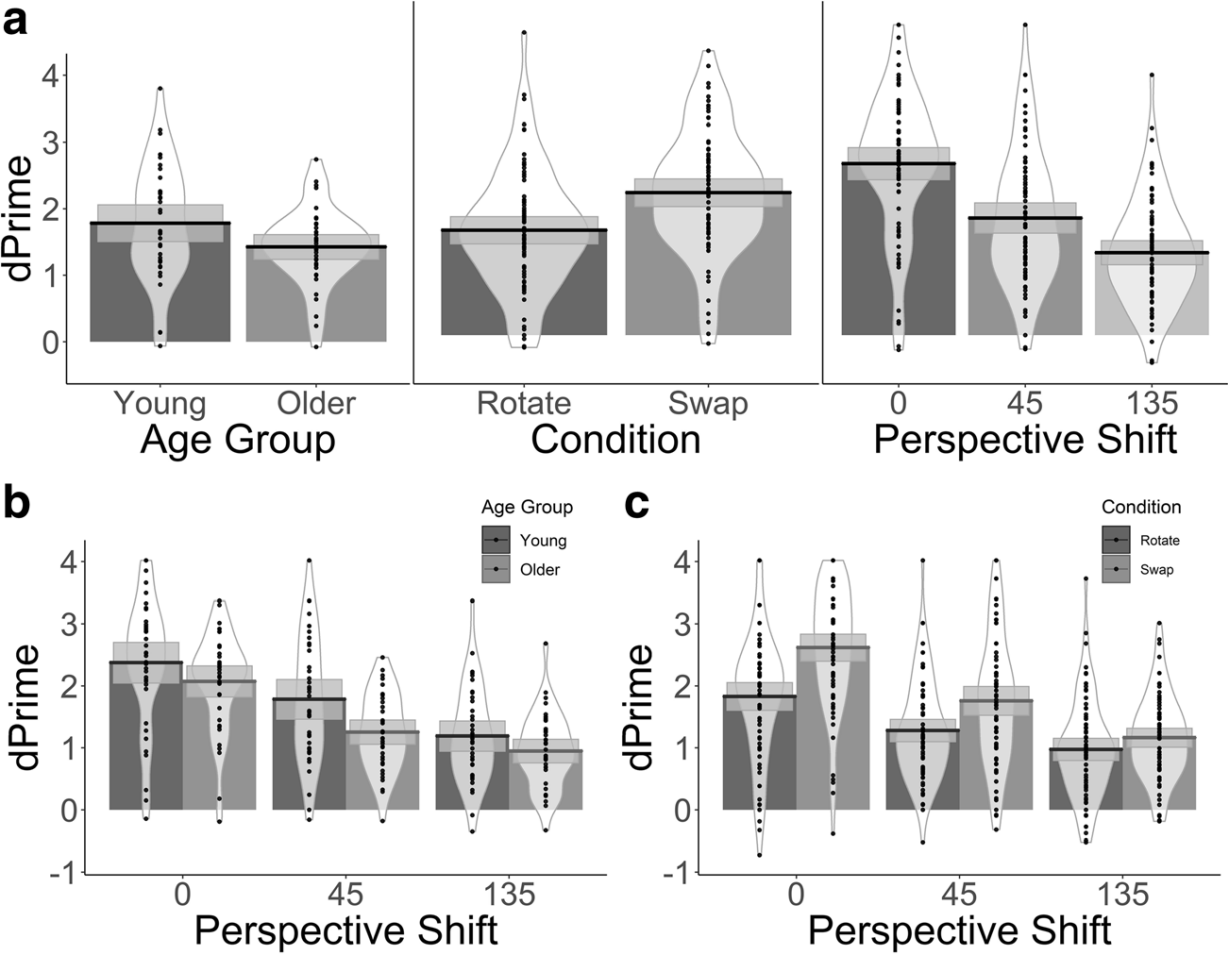
Bar plots for the d’ values with mean (solid line) and 95% CIs (grey-shaded area) with individual data points and violin plots. (**A**) Performance across Age Groups, Condition and Perspective Shift. (**B**) Younger and older participants’ as a function of Perspective Shift. (**C**) Performance in the *Swap* and *Rotate* conditions as a function of Perspective Shift

Estimates of sensitivity (d’) and bias (c) were obtained for each participant in the *Swap* and *Rotate* conditions and across the different perspective shifts (*0°, 45°, 135°*). Coefficients, standard errors and t-values are reported in Table 1 and show that Age Group, Perspective Shift and Condition were all reliable predictors of d’ scores (Fig. 2A). Specifically, we found a significant reduction in sensitivity in older adults when compared to younger adults. Perspective shifts from both *0°* to *45°* and from *45°* to *135°* also resulted in a significant reduction in sensitivity. Overall, sensitivity was lower in the *Rotate* than in the *Swap* condition.

**Table 1:** Coefficients from d’ LME analysis

*Predictors*	dPrime		
	*Estimates*	*std. Error*	*t-value*
Intercept	1.604	0.085	18.887
Age Group	-0.179	0.085	**-2.106**
Condition (*Rotate*)	-0.243	0.027	**-9.081**
Perspective Shift (*0°* to *45°*)	-0.705	0.063	**-11.085**
Perspective Shift (*45°* to *135°*)	-0.450	0.062	**-7.295**
Age Group: Condition (*Rotate*)	-0.015	0.027	-0.542
Age Group: Perspective Shift (*0°* to *45°*)	-0.114	0.064	-1.796
Age Group: Perspective Shift (*45°* to *135°*)	0.144	0.062	**2.341**
Condition (*Rotate*): Perspective Shift (*0°* to *45°*)	0.154	0.040	**3.878**
Condition (*Rotate*): Perspective Shift (*45°* to *135°*)	0.145	0.040	3.642
Age Group: Condition (*Rotate*): Perspective Shift (*0°* to *45°*)	0.044	0.040	1.103
Age Group: Condition (*Rotate*): Perspective Shift (*45°* to *135°*)	-0.045	0.040	-1.119

Significant t values (|t|≥1.96) are shown in **bold type**

We also found a significant interaction between Age Group and Perspective Shift from *45°* to *135°* (Fig. 2B). There also was a trend towards significance for the interaction between Age Group x Perspective Shift at *0°*–*45°* degrees. Specifically, the decline in performance was lower in older adults when the perspective shift increased from *45°* to *135°.* Finally, there was an interaction between Condition and Perspective Shift (*0°*–*45°*) with a larger decline in performance for the *Swap* condition than the *Rotate* condition with the introduction of the *45°* perspective shift (Fig. 2C).

Bias analysis suggested that participants were more conservative in the *Rotate* condition than in the *Swap* condition. Moreover, participants were less conservative with the introduction and increase of the perspective shift. The LME analysis of bias is reported in Online Supplementary Materials.

### Diffusion modelling

#### Model fit

Models that were at *p* < .05 level indicated model misfit. We removed five participants, four from the older group and one from the younger group, who had at least one significant model. For the purposes of visual representation and statistical analysis the drift rates in the *No* *Change* condition were multiplied by -1, as the correct answer in the *No Change* condition was the opposite to that in the *Swap* and *Rotate* conditions.

##### Starting bias

We did not find a starting bias (*z*) in older adults (M = 0.48, p =.165), but there was a slight bias towards the *No Change* response in the younger group (M =0.47, p =.026). The differences in starting bias between age groups were not statistically significant (p =.77).

##### Non-decision response times

As expected, we did find that older adults had higher non-decision response times (*t0*) than younger adults (M*young* = 1.00 s and M*old* = 1.99 s, p<.001).

Coefficients, standard errors and t-values for the drift rate (*v*) and response conservativeness (*a*) values are reported in Table 2.

**Table 2:** Coefficients from drift rate (v) and response boundaries (a) LME analysis

*Predictors*	Drift rate			Response boundaries		
	*Estimates*	*std. Error*	*t-value*	*Estimates*	*std. Error*	*t-value*
(Intercept)	0.614	0.034	**18.149**	2.973	0.086	**34.410**
Age Group	-0.103	0.034	**-3.053**	0.246	0.086	**2.849**
Condition (*Rotate*)	-0.473	0.033	**-14.423**	0.063	0.043	1.452
Condition (*Swap*)	-0.215	0.033	**-6.551**	0.261	0.043	**6.040**
Perspective Shift (*0°*–*45°*)	-0.400	0.057	**-7.028**	0.968	0.075	**12.961**
Perspective Shift (*45*–*135°*)	-0.118	0.057	**-2.078**	0.357	0.075	**4.780**
Age Group: Condition (*Rotate*)	0.000	0.033	0.013	0.055	0.043	1.265
Age Group: Condition (*Swap*)	-0.004	0.033	-0.117	0.129	0.043	**2.994**
Age Group: Perspective Shift (*0°*–*45°*)	-0.030	0.057	-0.524	0.143	0.075	1.915
Age Group: Perspective Shift (*45°*–*135°*)	-0.001	0.057	-0.020	0.112	0.075	1.503
Condition (Rotate): Perspective Shift (0°–45°)	0.306	0.080	**3.802**	-0.428	0.106	**-4.049**
Condition (Swap): Perspective Shift (0°–45°)	0.222	0.080	**2.757**	-0.583	0.106	**-5.521**
Condition (*Rotate*): Perspective Shift (*45°*–*135°*)	0.166	0.080	**2.067**	-0.064	0.106	-0.602
Condition (*Swap*): Perspective Shift (*45°*–*135°*)	0.031	0.080	0.379	-0.188	0.106	-1.779
Age Group: Condition (*Rotate*): Perspective Shift (*0°*–*45°*)	0.103	0.080	1.276	-0.056	0.106	-0.529
Age Group: Condition (*Swap*): Perspective Shift (*0°*–*45°*)	0.086	0.080	1.075	-0.119	0.106	-1.127
Age Group: Condition (*Rotate*): Perspective Shift (*45°*–*135°*)	0.011	0.080	0.131	-0.083	0.106	-0.785
Age Group: Condition (*Swap*): Perspective Shift (*45°*–*135°*)	0.066	0.080	0.822	-0.054	0.106	-0.512

Significant t values (|t|≥1.96) are shown in **bold** **type**

##### Drift rate

We found that Age Group, Perspective Shift and Condition were all significant predictors for drift rate. Specifically, drift rate in our older participants was lower than in the younger participants. In addition, across both age groups there was a reduction in drift rate in the *Rotate* and the *Swap* condition compared to the *No Change* condition. We also found that the introduction (*0°*–*45°*) and the increase (*45°*–*135°*) of the perspective shift led to a reduction in drift rate, with the introduction of the perspective shift leading to a larger decline in drift rate.

**Fig. 3:**
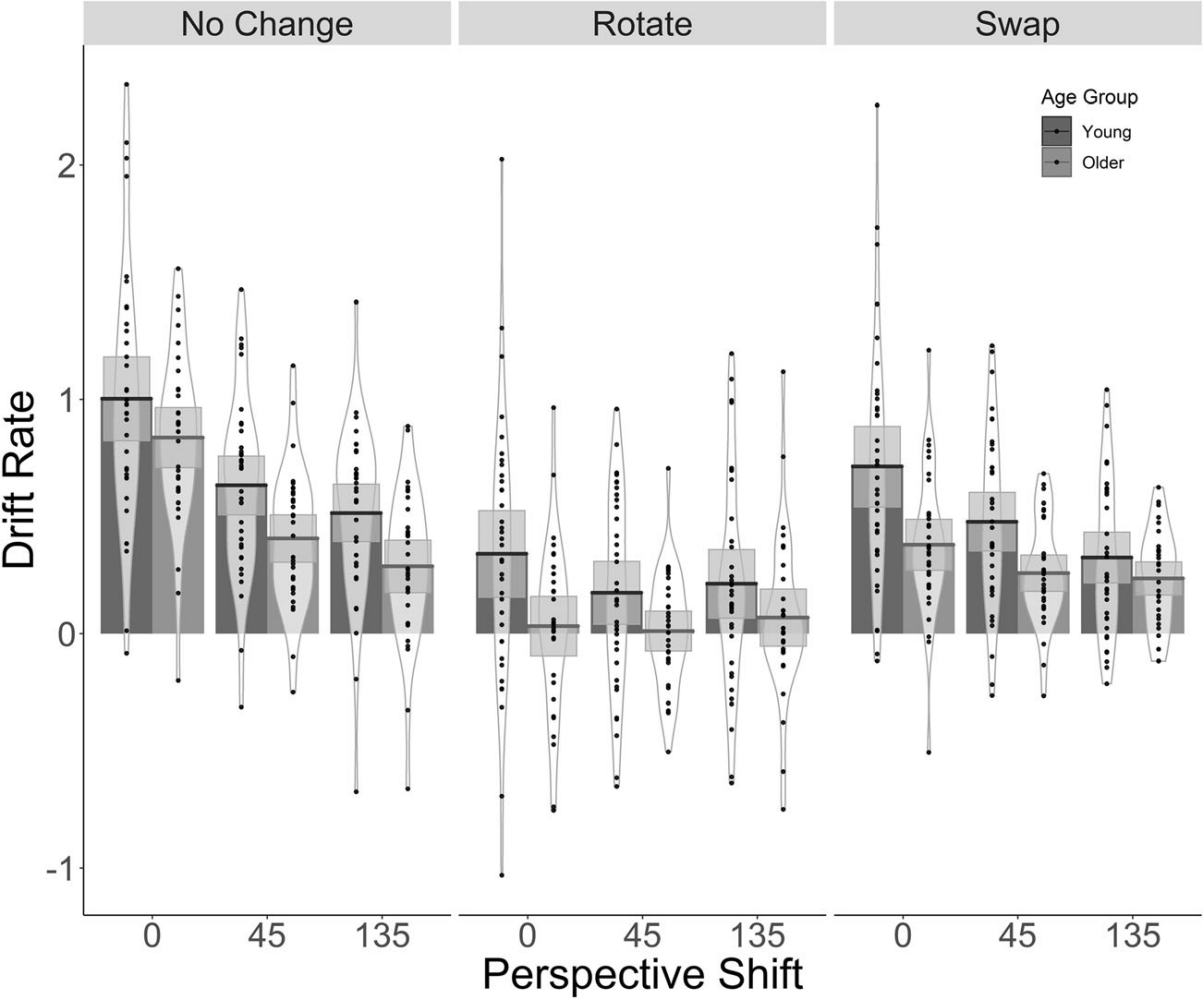
Bar plots for the drift rate values as a function of Perspective Shift, Condition and Age Group with mean (solid line) and 95% CIs (grey-shaded area) with individual data points and violin plots

The reduction in the drift rate was smaller in the *Rotate* and *Swap* conditions compared to the *No Change* condition when the perspective shift was introduced and when it increased from *45°* to *135°* in the *Rotate* condition*.* This is likely to be due to relative ease of the *No Change* condition when no perspective shift is present (see Fig. 3).

##### Response boundaries

We found main effects of Age Group, Condition and Perspective Shift on response boundaries. Consistent with previous research using diffusion modelling in ageing (Starns & Ratcliff, 2010), older adults had wider response boundaries, indicating that they needed to accumulate more information before making a decision and, as a result, took longer to make the decision. We also found that the response boundaries were wider in the *Swap* condition compared to the *No Change* condition. The introduction of perspective shift (*0°* vs. *45°*) led to a substantial widening of the response boundaries. A lesser increase was observed when perspective shift was further increased from *45°* to *135°*. We also found that older adults’ response boundaries increased in the *Swap* as compared to the *No Change* condition. There also was a trend for an interaction between Age Group and Perspective Shift (t = 1.92), whereby older adults response boundaries showed a larger increase compared to younger adults when the perspective shift was introduced (*0°*–*45°*). The increase in the response boundaries was smaller in the *Swap* and *Rotate* conditions compared to *No Change* when the perspective shift was introduced (*0°*–*45°*).

### Eye-tracking results

The aim of the eye-tracking analysis was to investigate age differences in encoding strategies and was therefore limited to the encoding phase.

#### General saccade and fixation parameters

Looking at general saccade and fixation parameters, we found differences between young and older age groups in saccade frequency, saccade average velocity, saccade peak velocity, saccade amplitude and saccade duration as well as fixation duration and fixation frequency (Table 3). Specifically, older adults made more saccades and of higher in velocity and amplitude. They also made more, but shorter, fixations compared to the younger adults. Similar results were observed when trials were split into correct and incorrect trials (see Online Supplementary Materials). There were no differences in blink frequency between the groups. Although these results suggest that older and younger adults were using different gaze strategies when encoding the stimuli, it is rather difficult to deduce the nature of these strategies from these general eye-tracking measures.

**Table 3:** Means and inferential statistics for saccade and fixation parameters between younger and older adults from the Learning Phase

Gaze measure	Mean young	Mean older	Confidence Interval	t-value	p-value
Saccade frequency	2.94	3.80	[-1.15, -0.57]	-5.52	**<.001**
Average velocity	100.94	110.68	[-16.74, -2.75]	-2.66	**.007**
Peak velocity	180.60	214.62	[-53.60, -14.46]	-3.40	**.003**
Amplitude	3.86	4.49	[-1.06, -0.19]	-2.76	**.007**
Saccade duration (ms)	32.48	34.95	[-4.87, -0.07]	-2.07	**.046**
Fixation frequency	3.15	4.08	[-1.24, -0.63]	-6.08	**<.001**
Fixation duration (ms)	325.33	270.15	[31.89, 78.48]	4.82	**<.001**
Blink frequency	0.44	0.38	[-0.06, 0.18]	0.96	.328

Significant p values are shown in **bold type**

**Fig. 4:**
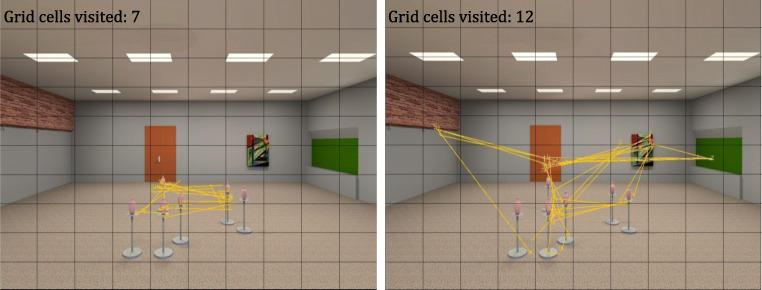
Trial examples with participant scan paths in a single trial with corresponding number of grid cells visited

Therefore, to further explore the differences in gaze characteristics between age groups and to develop a better understanding of how these relate to encoding strategies, we visually inspected the gaze paths for a random subset of the trials. This exploration suggested that our older adults tended to ‘look around more’, while the younger participants focused more on the target objects (see Fig. 4 for examples of gaze paths). There was substantial overlap of objects in the stimulus set used in this study, which made the stimuli unsuitable for interest-area analysis. For a post hoc analysis aiming to capture and quantify these observed differences and to compare gaze behaviour across different stimuli, we used a stimulus-independent grid cell measure.

#### Grid cell measure

To quantify the proportion of the stimulus that was examined during a trial, we superimposed a 10 x 10 grid on the stimulus display (Fig. 4). For each trial, we then calculated the total number of grid cells that received at least one fixation similar to the method used in Livingstone-Lee et al. (2011). We found that older adults examined a larger proportion of the display (M = 12.06) compared to younger adults (M = 10.12); t = -5.60, p = <.001, CI = [-2.62, -1.27]. Note that both age groups fixated only on a relatively small proportion of the display (10.12% and 12.06%, respectively). The fact that younger participants can perform the memory task better than the older participants while at the same time viewing less of the overall stimulus indicates that they were better at identifying the features within the display that were important for solving the task.

#### Gaze behaviour across the experiment

We also investigated if gaze behaviour changed across the experiment by correlating the number of grid cells visited with trial number for younger and older participants. There was a large negative correlation in younger (r = -.74, p < .001) but not in older participants (r = -.01, p = .621), suggesting that younger participants adapted their gaze strategy and explored less of the stimuli over the course of the experiment whilst older participants’ gaze behaviour did not change. The correlation coefficients between younger and older adults were statistically different (z = -9.13, p < .001).

#### Partial-correlation analysis

To investigate whether differences in the number of grid cells visited during encoding were, in fact, associated with performance, we ran partial correlations between drift rates and sensitivity (d’) with the number of grid cells visited, partialling out chronological age. There were no significant correlations between drift rate and the number of grid cells visited (r = -.18, p = .166) or between d’ and the number of grid cells visited (r = -.11, p = .383).

However, given the differences between the *Rotate* and *Swap* conditions in the behavioural findings, it is possible that the relationship between the number of grid cells visited and drift rate or d’ might be different across those two conditions. We, therefore, ran partial correlations separately for the *Rotate* and *Swap* conditions and found a significant correlation between the number of grid cells visited and drift rate in the *Rotate* condition (r = -0.29, p = .022), but not in the *Swap* condition (r = -0.13, p = 0.339). Similarly, there was a trend for a negative correlation between number of grid cells visited and d’ in the *Rotate* condition (r = -.22, p = .070) but not in the *Swap* condition (r = .02, p = .885).

## Discussion

In this study we used eye-tracking and diffusion modelling to investigate age-related changes in spatial memory for object locations. To ensure that the task did indeed address spatial memory and could not simply be solved by image comparisons, we introduced perspective shifts in two-thirds of the trials (Nardini, Thomas, Knowland, Braddick, & Atkinson, 2009). To investigate potential age-related differences in the resolution of spatial representations, we changed the spatial configuration between encoding and test by either swapping clusters of objects (coarse change) or by rotating a cluster within a scene (fine-grained change).

As expected, and in line with earlier research, we found that older adults had overall greater difficulties with the task than younger adults (cf. Hartley et al., 2007; Montefinese et al., 2015; Muffato et al., 2019), which was reflected in performance and drift rate differences between age groups. We also found that older adults were generally more conservative in their decision making and needed to accumulate more information prior to deciding on a response. The introduction of perspective shifts between encoding and test negatively affected performance in both age groups. Performance and drift rates were lower in the *Rotate* condition, which required more fine-grained spatial representations than the *Swa*p condition. In addition, both age groups became more conservative with the introduction of a perspective shift and in the *Swap* condition, but this effect was more pronounced in older adults. We also found differences in gaze behaviour between younger and older adults, suggesting differences in encoding strategies.

The lower sensitivity to detect changes and the lower drift rates in older adults suggest that they had greater difficulty in detecting whether or not object positions within the room had changed. These results are in line with previous research demonstrating age-related deficits in memory for layouts of objects or environmental features experienced from different perspectives during encoding and recall (Hartley et al., 2007; Montefinese et al., 2015; Muffato et al., 2019). Given that the target objects were present in learning and test, it is likely that age-related reductions in performance were in part driven by an inability to successfully bind the objects in the array to their specific locations (Muffato et al., 2019). The current study builds on previous research and suggests that an age-related decline in object-location binding is not mediated by the presence or absence of visual and geometrical cues (Muffato et al., 2019). The decline in older adults’ performance can be explained by age-related functional and morphological changes in the hippocampal circuit (Antonova et al., 2009; Meulenbroek et al., 2004; Moffat et al., 2007), which is crucial for development of spatial memories and manipulation of spatial memories to allow for perspective taking (King et al., 2002) as well as object-location binding (Postma & van der Ham, 2016; Zimmermann & Eschen, 2017).

To the best of our knowledge, the current study is the first to apply diffusion modelling to investigate age-related changes in spatial memory. Previously, diffusion modelling was mostly used to analyse data from relatively fast and simple reaction-time tasks, such as lexical decision or letter discrimination tasks (Ratcliff et al., 2004a, b; Thapar, Ratcliff, & McKoon, 2003). Our findings, consistent with Lerche and Voss (2019), suggest that diffusion modelling can also be used to study decision making in more complex tasks with longer response times. The observed age-related shift towards a more conservative response strategy is consistent with research that used diffusion modelling to study cognitive ageing across a number of different domains, including memory (Ratcliff, Gomez, et al., 2004a; Spaniol et al., 2006), perceptual learning (Ratcliff, Thapar & McKoon, 2006) and language (Ratcliff, et al., 2004a). Thus, it appears that this age-related shift towards a more conservative response strategy is not task/domain-specific but extends across several cognitive domains and tasks including those related to spatial memory. This shift is likely to be driven by emphasis on different aspects of the task between younger and older adults, with older adults being less accepting of errors at the expense of time (cf. Starns & Ratcliff, 2010).

Notably, older adults were not only more conservative in their responses, but also had longer non-decision response times. This could be due to slower visual encoding in older adults, driven by age-related declines in visual function (Owsley, 2011) and reduced motor speed (Ren et al., 2013). These findings highlight the importance of distinguishing information processing from decisional style and non-decisional components when analysing response-time data when studying cognitive ageing as age-related changes were evident in all these components. Together these components may explain the overall increase in response times in older adults during spatial-perspective taking reported in previous research (Watanabe, 2011; Watanabe & Takamatsu, 2014). In addition, we did not find starting bias in older adults, suggesting that older participants did not exhibit pattern-completion bias in the current task (Vieweg, Stangl, Howard, & Wolbers, 2015).

Unlike previous research in other cognitive domains that used diffusion modelling to study cognitive ageing (Ratcliff et al., 2004a, 2006a, b), we found an age-related decline in drift rate. Note, however, that the tasks used in earlier studies typically have only minimal memory demands and examine very different cognitive mechanisms such as lexical decision making or perceptual discrimination. Given that the introduction of a perspective shift equally affects younger and older subjects, age-related deficits in spatial-perspective taking abilities are unlikely to explain lower drift rates in older adults. Instead, we interpret the lower drift rates in our study as evidence of a reduced ability of our older adults to extract useful information from both the test stimuli and the stored representation (obtained during encoding) required to solve the task. As drift rates in the current task are reflective of the quality of the stored representation, the ability to compare it to the test stimuli, it is plausible that formation of an impoverished representation during encoding contributes to the observed lower drift rates. This idea is consistent with Ratcliff et al. (2004a), who interpreted drift rates as evidence of the quality of the memory trace for studied items in a recognition memory task. Given this interpretation of drift rates, lower drift rates in ageing are indicative of a specific spatial-processing deficit in ageing.

In line with previous research (Montefinese et al., 2015; Muffato et al., 2019; Watanabe, 2011), we observed performance declines with the introduction of a perspective shift in both age groups. These findings suggest that the 0° condition is qualitatively different from the conditions with a perspective shift. Specifically, the task in the 0° condition can be solved by accessing the learning scene from memory and using image matching to detect changes (Milner & Goodale, 2008; Nardini et al., 2009). However, when a perspective shift is introduced, the task becomes a spatial-perspective taking task that cannot be ‘simply’ solved by image matching. Instead, additional mental transformation of the stored spatial configuration to match the perspective at test with that of encoding (Hegarty & Waller, 2004) are required. These additional transformations are likely to recruit further brain regions, including the hippocampus circuit, which is associated with spatial processing (Mellet et al., 2000; Shelton & Gabrieli, 2002). Importantly, the performance and drift rate decline following the introduction of a perspective shift (i.e. from 0° to 45°) was almost three times larger than the decline observed when the perspective shift increased from 45° to 135°. These results suggest that it is the initiation of these mental transformations rather than the amount by which the spatial representations need to be transformed that produces the higher cognitive cost. Interestingly, this interpretation is inconsistent with findings from mental rotation research, which show that cognitive costs increase with increasing angular disparity, typically resulting in a linear increase in response times (Lohman, 1986; Shepard & Metzler, 1971). As we did not find a linear decrease in performance it is unlikely that our participants rotated the array to solve the tasks. Instead, they were more likely to imagine moving around the array to either match the test viewpoint with the encoded viewpoint or vice versa (King et al., 2002).

Participants in both age groups adopted a more conservative response strategy in trials in which the perspective shift was introduced and there was a trend for this increase to be higher in older adults. In addition, further increases in perspective shift resulted in adoption of even more conservative response strategies across both age groups. It is not surprising that participants have wider decision boundaries when a perspective shift is introduced, as they need to accumulate extra information to inform them about their new orientation. In addition, after participants accumulate information about the new orientation, they need to perform extra mental computations (Holmes et al., 2018), which come with an increased cognitive cost, to transform their stored representation of object locations to be consistent with that new perspective, and this additional cognitive demand is reflected in lower drift rates. Those results highlight that the spatial perspective shift not only increases processing demands but that it induces changes in response strategies, which are differentially affected by ageing. This is particularly important for research on spatial-perspective taking that frequently relies on measures of response times as a marker of performance (i.e. Spatial Orientation Test; Guilford & Zimmerman, 1948; Hegarty & Waller, 2004).

Results of previous research on the effects of ageing on spatial-perspective taking are mixed (Montefinese et al., 2015; Muffato et al., 2019; Watanabe, 2011). If there is an age-related spatial-perspective-taking deficit, we expected to find an age-by-perspective interaction. Although we did find an interaction, it was not of the form we expected. Specifically, we found that performance in older adults did not decline as much as it did in younger participants when the perspective shift was increased from 45° to 135°; this is consistent with Montefinese et al.’s (2015) findings. We believe that this interaction was driven by older adults being more affected by the introduction of a perspective shift (interaction approaching significance). This contrasts with the performance of the younger group, suggesting that the younger group was better able to deal with the introduction of a perspective shift as they showed a more linear decline in performance with the increasing size of the perspective shifts, which at 135° almost matched the performance of the older adults group. Therefore, the larger drop in performance in older adults with the introduction of the perspective shift and no decline in performance with the increase of the perspective shift suggests that ageing may be affecting the initiation of the extra mental computations that are required for spatial-perspective taking. In addition, the age-by-perspective interaction may arise due to floor performance. That is, it is possible that older adults perform at floor levels when the perspective shift is introduced, and their performance thus remains unchanged with the increase in the perspective shift

Our results show that the *Rotate* condition was more difficult than the *Swap* condition. This was expected as the *Swap* condition, but not the *Rotate* condition, could be solved with a coarse spatial representation. Specifically, the *Swap* condition can be solved by representing the spatial relationships between the object clusters or the coarse locations of the object clusters in the room. The *Rotate* condition, in contrast, also requires participants to encode the precise orientation of each object cluster either relative to the other clusters or relative to the room. This additional difficulty in the *Rotate* condition is reflected in substantially lower drift rates, which suggests that participants found it more difficult to extract useful information to identify a change in object positions when comparing the memory trace formulated during encoding to the position of objects at test. Surprisingly, we found that participants were more conservative in the *Swap* than in the *Rotate* condition. One possible explanation for this effect is that participants preferred to accumulate more information in the *Swap* condition, thus increasing the likelihood of producing correct answers. In contrast, in the *Rotate* condition, extracting useful information was more difficult (reflected in low drift rates), and spending additional time would not necessarily lead to any substantial information gain. This explanation may also apply to the Age Group and Condition interaction, in which older adults’ response boundaries were wider in the *Swap* condition.

One of the aims of this study was the examination of age-related differences in spatial-encoding strategies using eye tracking and how those potential strategy differences are related to performance differences. We first examined general gaze patterns during encoding and found that older adults made more saccades than younger participants that were larger in amplitude and velocity (peak and average) and longer in duration. They also made more fixations that were consequently shorter in duration as they are bound by fixed encoding times. These patterns are not reflective of previous ageing research using other tasks that have reported that ageing is associated with reductions in saccade amplitudes, velocity and frequency (Dowiasch, Marx, Einhäuser, & Bremmer, 2015; Hilton, Miellet, Slattery, Wiener, 2019; Porter et al., 2010; Williams, Zacks, & Henderson, 2009). Consistent with our findings, Açik, Sarwary, Schultze-Kraft, Onat, and König (2010) found that older adults made more fixations when viewing complex visual stimuli. However, they also reported that saccade amplitudes were lower in older adults. One explanation for age-related declines in saccade amplitudes along with an increased fixation count is that the size of useful field of view declines in older age, resulting in an increased number of fixations that are closer to each other (Sekuler, Bennett, & Mamelak, 2000). This account does not, however, explain our findings as older adults produced saccades with larger amplitudes. We thus believe that the differences in these general parameters in this study reflect differences in encoding strategies rather than resulting from the general ageing of the oculomotor system.

In the current task, the environment contained room-based cues and room geometry. This contrasts with Muffato et al.’s (2019) study where objects were presented in an open-field and object locations could only be remembered by encoding the spatial relationships between the objects, whilst in our task participants could use different encoding strategies to encode object locations. Specifically, participants could either encode locations by focusing on the spatial relations among object clusters or by relating the object positions to other cues. Adoption of the latter strategy may be reflected in the gaze data as participants would presumably fixate on the objects as well as on the environmental cues.

To further explore how age differences in general gaze patterns might translate to differences in spatial encoding strategies, we looked at the percentage of the stimulus attended to during encoding. Specifically, we found that older adults examined more of the stimuli. We interpret these findings as indicative of older adults employing an encoding strategy in which they tend to remember target object positions in relation to room-based cues, while younger adults focus on the spatial relationship between object clusters.

An alternative explanation for why older adults were looking at room-based cues is that they were distracted by their presence. This is consistent with a prominent theory of cognitive ageing stating that older adults have difficulty in inhibiting attention to salient but task-irrelevant stimuli (Hasher & Zacks, 1988). The current design does not allow us to differentiate between those two alternative explanations as the stimulus set was not suited for interest-area analyses. We are, however, currently running further experiments to distinguish between these alternative explanations. Preliminary analyses of these experiments suggest that older adults rely on extra cues to facilitate encoding (Segen et al., in preparation). To further investigate age-related differences in encoding strategies, future research could also make use of verbal reports during encoding or retrospective strategy reports. Such approaches may shed light on whether older adults explicitly adapt their encoding strategies to compensate for spatial memory deficits.

Interestingly, we found a negative correlation between the percentage of stimuli attended to and drift rate, but only in the *Rotate* condition. Our conjecture is that participants who explored a smaller proportion of the stimuli were more efficient at sampling the parts of stimuli that were most informative for formulating the fine-grained representations required to solve the task in this condition. The higher drift rate in the *Rotate* condition is in line with this explanation. However, in situations in which a coarser representation is sufficient, relating target objects to environmental cues is sufficient to solve the task. As already noted, older adults were more likely to look around more during encoding, which could be indicative of coarser spatial encoding. Adoption of such an encoding strategy would have enabled them to solve the *Swap* condition but not the *Rotate;* this interpretation is consistent with our diffusion-modelling results as the drift rates are around zero for older adults in the *Rotate* condition and are slightly higher in the *Swap* condition. Drift rates around zero imply that older participants are sampling from a largely uninformative representations in the *Rotate* condition, whilst the positive drift rates in the *Swap* condition are indicative of ability to extract some useful information from the comparison between the stored representation formed during encoding and test stimuli to detect if the spatial arrangement has changed. In addition, we also found that in younger participants gaze became more focused over the course of the experiment whilst in older adults gaze remained consistent throughout the experiment. We believe this adaptation of gaze behaviour in our young participants reflects their ability to improve their encoding strategy with practice.

Overall, our exploratory eye-tracking analyses suggest that spatial representations useful for the task presented here can be enhanced by adopting a visual-encoding strategy that involves focusing on the to-be-encoded objects. This interpretation is consistent with research showing that focal shifts of spatial selective attention to the memorised locations is associated with active maintenance of location-specific representations within visuo-spatial working memory (Awh, Jonides, & Reuter-Lorenz, 1998; Shimi & Scerif, 2017; Smyth & Scholey, 1994). Thus, by focusing on the to-be-remembered objects participants are more likely to maintain location-specific representations within their visuo-spatial working memory. This encoding behaviour is likely to contribute to the formation of a stronger long-term memory trace that participants can access at test (Ranganath, Cohen, & Brozinsky, 2005). Young participants were more likely to adopt this strategy during encoding, which could explain higher performance in our younger adults’ group. However, those interpretations would benefit from further investigation as the reported correlations were explorative in nature and yielded relatively small effects.

In summary, we have presented a novel task to investigate age-related differences in the ability to encode spatial relationships between objects and to recognize them across different viewpoints. As expected, we found that older adults performed worse than younger participants on the task, and overall participants found the condition that required more fine-grained spatial representations harder than the condition that could be solved using a coarser representation. We also found that older adults’ encoding strategies differed from those of younger participants. Moreover, the differences in encoding strategies identified via eye-movement behaviour were correlated with performance differences across different manipulations. This highlights the value of using eye movements to study tasks involving the memory of visual scenes. Our diffusion-modelling analysis shows that declines in spatial memory are likely to be driven by specific declines in spatial processing rather than general age-related declines in cognition, whilst also highlighting that an age-related shift towards a more conservative response strategy appears to extend across a wide range of cognitive tasks.

### Open practices statement

The datasets generated during and/or analysed during the current study are available in the Open Science Framework repository, https://osf.io/xh5kd/. This experiment was not preregistered.

## Electronic supplementary material

ESM 1 (DOCX 23 kb)
