# Supplementary material for: Age-related differences in visual encoding and response strategies contribute to spatial memory deficits
Source: Mem Cognit. 2020 Aug 31;49(2):249–64. doi: 10.3758/s13421-020-01089-3 (PMC7886755; doi:10.3758/s13421-020-01089-3)
Supplement: Supplementary file 1 — (DOCX 23 kb) [file 13421_2020_1089_MOESM1_ESM.docx]

LME Analysis with Sex as a Factor

Table 1 Coefficients from d’ LME analysis

|  | **dPrime** | | |
| --- | --- | --- | --- |
| *Predictors* | *Estimates* | *std. Error* | *t-value* |
| Intercept | -0.155 | 0.120 | -1.289 |
| Age Group | 0.400 | 0.169 | 2.373 |
| Condition (*Rotate*) | -0.274 | 0.039 | -7.043 |
| Perspective (*0°* to *45°*) | -0.800 | 0.093 | -8.630 |
| Perspective (*45°* to *135°*) | -0.315 | 0.092 | -3.442 |
| Sex *(Female)* | -0.092 | 0.120 | -0.769 |
| Age Group: Condition (*Rotate*) | 0.049 | 0.055 | 0.887 |
| Age Group: Perspective (*0°* to *45°*) | 0.238 | 0.130 | 1.828 |
| Age Group: Perspective (*45°* to *135°*) | -0.279 | 0.129 | -2.168 |
| Condition (*Rotate*): Perspective (*0°* to *45°* ) | 0.196 | 0.059 | 3.318 |
| Condition (*Rotate*): Perspective (*45°* to *135°* ) | 0.091 | 0.059 | 1.539 |
| Age Group: Sex *(Female)* | -0.224 | 0.169 | -1.326 |
| Condition (*Rotate*): Sex *(Female)* | 0.065 | 0.039 | 1.671 |
| Perspective (*0°* to *45°* ): Sex *(Female)* | -0.072 | 0.093 | -0.779 |
| Perspective (*45°* to *135°* ): Sex *(Female)* | 0.036 | 0.092 | 0.397 |
| Age Group: Condition (*Rotate*): Perspective (*0°* to *45°* ) | -0.090 | 0.083 | -1.086 |
| Age Group: Condition (*Rotate*): Perspective (*45°* to *135°* ) | 0.103 | 0.083 | 1.238 |
| Age Group: Condition (*Rotate*): Sex *(Female)* | -0.077 | 0.055 | -1.404 |
| Age Group: Perspective (*0°* to *45°* ): Sex *(Female)* | -0.064 | 0.130 | -0.491 |
| Age Group: Perspective (*45°* to *135°* ): Sex *(Female)* | -0.038 | 0.129 | -0.292 |
| Condition (*Rotate*): Perspective (*0°* to *45°* ): Sex *(Female)* | 0.010 | 0.059 | 0.168 |
| Condition (*Rotate*): Perspective (*45°* to *135°* ): Sex *(Female)* | 0.037 | 0.059 | 0.627 |
| Age Group: Condition (*Rotate*): Perspective (*0°* to *45°* ): Sex *(Female)* | 0.013 | 0.083 | 0.153 |
| Age Group: Condition (*Rotate*): Perspective (*45°* to *135°* ): Sex *(Female)* | -0.055 | 0.083 | -0.660 |

Significant t values (|t|≥1.96) in **bold**

LME analysis for Bias

Table 1 Coefficients from Bias LME analysis

|  | **Bias** | | |
| --- | --- | --- | --- |
| *Predictors* | *Estimates* | *std. Error* | *t-value* |
| Intercept | 0.026 | 0.047 | 0.558 |
| Age Group | -0.053 | 0.067 | -0.789 |
| Condition (*Rotate*) | 0.129 | 0.019 | **6.805** |
| Perspective (*0°* to *45°*) | -0.358 | 0.055 | **-6.544** |
| Perspective (*45°* to *135°*) | -0.185 | 0.050 | **-3.702** |
| Age Group: Condition (*Rotate*) | -0.014 | 0.027 | -0.542 |
| Age Group: Perspective (*0°* to *45°*) | 0.217 | 0.077 | **2.806** |
| Age Group: Perspective (*45°* to *135°*) | 0.127 | 0.071 | 1.797 |
| Condition (*Rotate*): Perspective (*0°* to *45°*) | -0.099 | 0.028 | **-3.522** |
| Condition (*Rotate*): Perspective (*45°* to *135°*) | -0.050 | 0.028 | -1.784 |
| Age Group: Condition (*Rotate*): Perspective (*0°* to *45°*) | 0.044 | 0.040 | 1.103 |
| Age Group: Condition (*Rotate*): Perspective (*45°* to *135°*) | -0.045 | 0.040 | -1.119 |

Significant t values (|t|≥1.96) in **bold**

| **Table 1** Means and t-test results for saccade and fixation parameters between younger and older adults from the Learning Phase | | | | | |
| --- | --- | --- | --- | --- | --- |
| Gaze measure | Mean Young | Mean Older | Confidence Interval | t-value | p-value |
| **Correct trials** |  |  |  |  |  |
| Saccade Frequency | 2.93 | 3.79 | [-1.16,-0.56] | -5.65 | **<.001** |
| Average velocity | 100.61 | 110.47 | [-16.44, -3.27] | -2.83 | **.006** |
| Peak velocity | 180.03 | 213.69 | [-54.11, -13.20] | -3.29 | **.003** |
| Amplitude | 3.85 | 4.47 | [-1.05, -0.20] | -2.80 | **.008** |
| Saccade duration (ms) | 32.42 | 34.93 | [-4.89, -0.12] | -2.08 | **.041** |
| Fixation Frequency | 3.14 | 4.08 | [-1.23, -0.65] | -6.49 | **<.001** |
| Fixation Duration (ms) | 325.85 | 270.17 | [33.26, 78.09] | 5.01 | **<.001** |
| Blink Frequency | 0.38 | 0.43 | [-0.06, 0.18] | -0.96 | .326 |
| **Incorrect Trials** |  |  |  |  |  |
| Saccade Frequency | 2.98 | 3.82 | [-1.21, -0.48] | -4.66 | **<.001** |
| Average velocity | 102.09 | 111.43 | [-17.22, -1.47] | -2.29 | **.020** |
| Peak velocity | 182.80 | 217.53 | [-53.33, -16.13] | -3.75 | **.001** |
| Amplitude | 3.90 | 4.56 | [-1.30, -0.18] | -2.65 | **.011** |
| Saccade duration (ms) | 32.60 | 35.06 | [-1.13, -.018] | -2.65 | **.011** |
| Fixation Frequency | 3.16 | 4,10 | [-1.24, -0.63] | -6.01 | **<.001** |
| Fixation Duration (ms) | 323.37 | 268.93 | [31.78, 77.15] | 4.89 | **<.001** |
| Blink Frequency | 0.39 | 0.45 | [-0.19, 0.07] | -0.98 | .315 |
| ***Note: significant p values are in bold*** | | | | | |

Gaze Parameters for Correct and Incorrect Trials
